# Accurate modeling of temporal correlations in rapidly sampled fMRI time series

**DOI:** 10.1002/hbm.24218

**Published:** 2018-06-08

**Authors:** Nadège Corbin, Nick Todd, Karl J. Friston, Martina F. Callaghan

**Affiliations:** ^1^ Wellcome Centre for Human Neuroimaging, UCL Institute of Neurology, University College London London United Kingdom; ^2^ Department of Radiology Harvard Medical School, Brigham and Women's Hospital Boston Massachusetts

**Keywords:** accelerated acquisitions, functional MRI, functional sensitivity, prewhitening, temporal correlation

## Abstract

Rapid imaging techniques are increasingly used in functional MRI studies because they allow a greater number of samples to be acquired per unit time, thereby increasing statistical power. However, temporal correlations limit the increase in functional sensitivity and must be accurately accounted for to control the false‐positive rate. A common approach to accounting for temporal correlations is to whiten the data prior to estimating fMRI model parameters. Models of white noise plus a first‐order autoregressive process have proven sufficient for conventional imaging studies, but more elaborate models are required for rapidly sampled data. Here we show that when the “FAST” model implemented in SPM is used with a well‐controlled number of parameters, it can successfully prewhiten 80% of grey matter voxels even with volume repetition times as short as 0.35 s. We further show that the temporal signal‐to‐noise ratio (tSNR), which has conventionally been used to assess the relative functional sensitivity of competing imaging approaches, can be augmented to account for the temporal correlations in the time series. This amounts to computing the *t*‐score testing for the mean signal. We show in a visual perception task that unlike the tSNR weighted by the number of samples, the *t*‐score measure is directly related to the *t*‐score testing for activation when the temporal correlations are correctly modeled. This score affords a more accurate means of evaluating the functional sensitivity of different data acquisition options.

## 1 INTRODUCTION

Functional magnetic resonance imaging (fMRI) relies on the validity of statistical tests to make reliable inference about neuronal activity underlying the experimentally observed blood‐oxygen‐level‐dependent (BOLD) effect. Increasing the number of samples is an effective way of increasing statistical power. The emergence of advanced MRI acquisition strategies such as parallel (Griswold et al., 2002; Pruessmann, Weiger, Scheidegger, & Boesiger, 1999) and multiband (or simultaneous multi‐slice) imaging (Breuer et al., 2005; Larkman et al., 2001; Setsompop et al., 2012) have enabled sampling rates to be greatly increased by reducing the time taken to acquire a single volume. Reducing the volume acquisition time not only allows the acquisition of more samples within the same total duration but also reduces sensitivity to intravolume motion and improves the sampling of physiological noise, which can then be more effectively removed by low‐pass filtering the time series (Narsude, Gallichan, van der Zwaag, Gruetter, & Marques, 2016; Todd et al., 2017).

However, concurrent with these benefits are penalties that must also be considered—to determine the overall impact of a given rapid imaging protocol on functional sensitivity. Accelerated imaging techniques that capitalize on coil information to acquire less data—and subsequently unfold the resulting aliased images—are susceptible to varying degrees of noise amplification, depending on the geometry factor (g‐factor) of the coil and the k‐space sampling scheme used. The need to obtain calibration data characterizing the coil elements also increases sensitivity to intervolume motion; as such motion reduces spatial correspondence between the calibration data and the aliased images to be unfolded. With parallel imaging, a further signal‐to‐noise ratio (SNR) penalty results from the omission of k‐space samples, whereby an acceleration factor *R* reduces the SNR by a factor of $\sqrt{R}$. The shorter repetition time (TR) of multiband imaging and the concomitant reduction in flip angle, also reduces the steady‐state magnetization and therefore the SNR.

The focus of this work is the increase in serial or temporal correlation that results from increasing the sampling rate (Purdon & Weisskoff, 1998); for example, due to physiological effects, motion, or scanner drift. These correlations mean that the effective degrees of freedom are smaller than the number of acquired samples. Modeling temporal correlations is essential to preclude overestimation of functional sensitivity and to prevent an inflated false‐positive rate. A well‐established approach for modeling and removing temporal correlations is to use a mixture of white noise and a first‐order autoregressive model (Friston et al., 2002). However, with the advent of rapid imaging techniques, it has been shown that such a model may not sufficiently capture temporal correlations in time series acquired with very short TR (Bollmann, Puckett, Cunnington, & Barth, 2018; Eklund, Andersson, Josephson, Johannesson, & Knutsson, 2012; Olszowy, Williams, Rua, & Aston, 2017). To address this issue, a more complex model, implemented under the name of “FAST” in SPM12 (R7203, Wellcome Centre for Human Neuroimaging, http://www.fil.ion.ucl.ac.uk/spm) is investigated here. While the use of this method has previously been reported (Todd et al., 2016), to our knowledge, only one recent study (Bollmann et al., 2018) investigated its performance in terms of prewhitening. Here, we present a complementary analysis of the effectiveness of the approach for removing temporal correlations from rapidly sampled time series. The efficiency of the model is investigated with sampling intervals (volume TRs) ranging from 0.35 to 2.8 s.

The range of penalties and benefits associated with rapid imaging techniques make it difficult to predict which set of sequence parameters will provide the optimal functional sensitivity. As the aim of fMRI is to detect a BOLD‐related signal change over and above random fluctuations in the time series, a commonly used measure is the temporal signal‐to‐noise ratio (tSNR). This measure quantifies the mean signal relative to its standard deviation over time and therefore requires an accurate estimate of the standard deviation of the time series. However, the standard deviation will be underestimated, and therefore, the tSNR overestimated, if temporal autocorrelations are not taken into account and modeled appropriately. Even if the standard deviation is correctly estimated, the tSNR will still not account for the effective degrees of freedom afforded by the time series. To incorporate this important determinant of functional sensitivity (Murphy, Bodurka, & Bandettini, 2007), the estimated tSNR value has previously been multiplied by the square root of the number of samples (Smith et al., 2013), or (equivalently) divided by the square root of the TR (Poser, Koopmans, Witzel, Wald, & Barth, 2010). However, this approach assumes independent samples and will likely overestimate the functional sensitivity of a protocol, particularly when high temporal sampling rates are used. Here, we use the general linear model (GLM) framework, as typically used to analyze fMRI time series (Worsley & Friston, 1995), to make an inference about the mean signal contrast using a *t*‐score. Characterising a time series in this way ensures that the imaging protocol is evaluated within the same context as the detection of functional activation—and allows varying degrees of temporal correlation present in the data to be accounted for. We show that, with a well‐controlled number of parameters in the “FAST” model, this measure is directly related to the t‐score testing for functional activation in a visual perception experiment, unlike the conventionally used metrics. Therefore, with the proposed approach, the benefits of highly accelerated protocols can be more accurately quantified and compared in terms of functional sensitivity, even when the numbers of samples and temporal correlations present in the time series are varied.

## 2 THEORY: GLM/T‐SCORE

### 2.1 The GLM parameters estimation and *t*‐score computation

Detection of neuronal activity via the BOLD signal in fMRI is most often based on describing the acquired data, *Y*, by a GLM (Worsley & Friston, 1995):

(1) $$Y=X\beta +\epsilon \;\epsilon ∼N(0,\sigma^{2}V)$$

Each row of the design matrix, *X*, corresponds to a single observation (i.e., acquisition volume). Each column represents an explanatory variable, for which β constitutes the regression coefficients. ϵ is an error term, assumed to follow a Gaussian distribution with zero mean, and covariance $\sigma^{2}V$, where σ is the standard deviation and *V* is an autocorrelation matrix.

The most common approach for making inferences about neuronal activation in response to a task is based on classical statistics. Classical inference relies on calculating a *t*‐score to evaluate the significance of an effect of interest, $c^{T}\beta$:

(2) $$t=\frac{c^{T}\hat{\beta }}{\sqrt{var\left(c^{T}\hat{\beta }\right)}}=\frac{c^{T}\hat{\beta }}{\hat{\sigma }\sqrt{c^{T}X^{-}{VX}^{-T}c\;}}$$

Here, c is a contrast vector of weights and $\hat{\beta }=X^{-}Y$ are maximum likelihood estimates with “^−^” denoting the pseudoinverse.

However, if temporal autocorrelations are present in the time series (i.e., in the case of nonsphericity), such that *V* is not equal to the identity matrix, the denominator of Equation 2 will no longer be the square‐root of a χ^2^ distribution. In this case, Equation 2 no longer follows a *t*‐distribution, which prohibits inference based on comparing it to a Student t null distribution (Kiebel & Holmes, 2003).

One solution is to adjust the degrees of freedom of the Student *t* null distribution compared against, using the Satterthwaite approximation based on moment matching (Worsley & Friston, 1995). A second solution, which is used here, rests on whitening the data before fitting the GLM. The whitening matrix *W* is defined by $W^{T}W=V^{-1}$ . Equation 1 becomes

(3) $$WY=WX\beta +W\epsilon \;W\epsilon ∼N(0,\sigma^{2}I)$$

where *I* is the identity matrix. Both solutions require an accurate model of the temporal correlation within the time series to correctly estimate *V*. Once *V* is estimated and the data are whitened, the maximum likelihood regression coefficients $\hat{\beta }$ are estimated via ${\left(WX\right)}^{-}WY$ and the standard deviation of the error term $\hat{\sigma }$ is estimated from the residuals of the GLM fit. The *t*‐score now follows a Student *t*‐distribution and can then be calculated as follows:

(4) $$t=\frac{c^{T}\hat{\beta }}{\sqrt{var\left(c^{T}\hat{\beta }\right)}}=\frac{c^{T}\hat{\beta }}{\hat{\sigma }\sqrt{c^{T}{\left(WX\right)}^{-}{\left(WX\right)}^{-T}c\;}}=\frac{c^{T}\hat{\beta }}{\hat{\sigma }\eta }$$

The parameter η is introduced to illustrate the impact of the prewhitening step.

In this framework, the variance is estimated as proposed by Worsley and Friston (1995) according to Equation 5:

(5) $${\hat{\sigma }}^{2}=\frac{e^{t}e}{trace(RV_{w})}$$

where e=RWY are the residuals and *R* is the residual forming matrix $R=I-WX{\left(X^{T}W^{T}WX\right)}^{-1}X^{T}W^{T}$. $V_{w}$ is the autocorrelation matrix after prewhitening the data and therefore taken to be the identity matrix in this framework.

### 2.2 Model of temporal correlations for rapidly sampled data

Estimating the covariance matrix is a key element of the analysis since it is used to derive the prewhitening matrix *W*. The autocorrelation matrix *V* can be estimated, using Restricted Maximum Likelihood (ReML), as a linear combination of a fixed set of covariance components, $V=\sum_{i}\lambda_{i}C^{i}$ modeling a mixture of white noise and a first‐order autoregressive process AR(1) (Friston et al., 2002). However, recent studies have shown that this simple model with two components may not be enough to model temporal correlations of accelerated sequences with more rapid sampling rates (Bollmann et al., 2018; Eklund et al., 2012; Olszowy et al., 2017). To address this issue, a model of serial correlations comprised of an extended basis set of covariance matrices has been developed. This algorithm, termed “FAST,” is implemented as a processing option in SPM.

In this model, a dictionary of covariance components, of length 3*p*, is composed of *p* different exponential time constants, α (indexed by *q*) and their derivatives with respect to α up to second order (indexed by *n*) by constructing a set of Toeplitz matrices $C^{n\alpha }$ with elements defined as follows:

Cijnα= 1 if j=i and n=0j−ine−αj−i otherwise, n∈0,2

(6) $$with\;\alpha =\frac{8}{2^{q}}\;,\;q\in \left[1,p\right]$$

**Figure 1:**
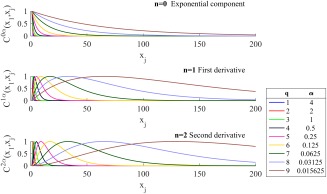
Covariance components of the FAST model with *p* = 9 [Color figure can be viewed at http://wileyonlinelibrary.com]

The covariance components included in this model are illustrated in Figure 1 for the case of *p* = 9.

### 2.3 Functional sensitivity measure accounting for temporal correlations

Considering a contrast vector, $c_{0}$, testing the mean signal, the resulting *t*‐score would be

(7) $$t_{0}=\frac{c_{0}^{T}\hat{\beta }}{\hat{\sigma }\sqrt{c_{0}^{T}{\left(WX\right)}^{-}{\left(WX\right)}^{-T}c_{0}\;}}=\frac{c_{0}^{T}\hat{\beta }}{\hat{\sigma }\eta_{0}}$$

This *t*‐score testing for the mean signal can be used to evaluate the functional sensitivity of the imaging approach with which the time series was acquired. In the simple case of *X* being a unitary vector of length *N*, and *W* being the identity matrix (i.e,. there being no temporal correlation in the data), the *t*‐score testing for the mean signal, $t_{0}$, reduces to the more commonly used tSNR weighted by the square root of the number of samples, as $\eta_{0}$ reduces to $\sqrt{\frac{1}{N}}$ . As such, $\eta_{0}$ can be viewed as the “inverse effective degrees of freedom” or the “effective precision.” In other words, this measure captures the uncertainty we have about our estimate ( $\hat{\sigma }$) of the standard deviation. Note that this is distinct from the degrees of freedom of the student distribution of the *t*‐score. In the presence of temporal correlations that are correctly modeled, this uncertainty estimate will accurately increase leading to an overall decrease in the *t*‐score testing for the mean signal. Conversely, if these temporal correlations are not modeled accurately, the effective precision of the variance estimate will be inflated (in other words, our uncertainty estimate will be inaccurately low), falsely increasing the *t*‐scores and producing an increased false‐positive rate.

In what follows, the *t*‐score testing for the mean signal $t_{0}$ and the weighted tSNR, labelled $tSNR_{w}$, are computed based on the GLM parameters: $t_{0}=\frac{c_{0}^{T}\hat{\beta }}{\hat{\sigma }\eta_{0}}$ and t $SNR_{w}=\frac{c_{0}^{T}\hat{\beta }}{\hat{\sigma }}\sqrt{N}$. Calculating the weighted tSNR via the GLM framework allows the task‐related variance to be removed via the design matrix regressors in the same way as for the *t*‐score testing for the mean signal. It will be shown that under the proviso of accurate modeling of temporal correlation, $t_{0}$ is a more accurate measure of functional sensitivity than $tSNR_{w}$.

## 3 METHODS

### 3.1 fMRI data acquisition

fMRI time series data were acquired from ten healthy volunteers (aged between 25 and 45 years, 7 females), with approval granted by the local ethics committee of the institution and the informed written consent of the participants. The task consisted of passive viewing of images of scenes and objects and a baseline condition in which participants viewed a thin white circle on a gray background. Scenes or objects were displayed for 2 s each during a block of 8 s, followed by a baseline block of 8 s. Blocks were separated by an interval varying between 2 and 4 s. In order to maintain attention, participants were instructed to count the number of times that the white circle flashed during the baseline condition and report whether the number of flashes was odd or even via a button press at the end of each baseline block. A total of 60 scene images and 60 object images were presented over each 7 min run. The task was repeated 4 times for each participant with a different multiband (MB) factor for each run. The order of the different MB factors was counterbalanced across participants.

The data were acquired on a 3 T Tim Trio (Siemens, Erlangen, Germany) using a 2D gradient echo EPI sequence with multiband capability for simultaneous excitation of multiple slices (R012, from the Center for Magnetic Resonance Research, University of Minnesota). This sequence utilizes the blipped‐CAIPI approach for controlled aliasing of simultaneously excited slices (Setsompop et al., 2012). Sequence parameters were chosen to be similar to those typically used for moderate resolution whole‐brain fMRI studies at 3 T and are summarized in Table 1. Multiband (MB) factors of 1, 2, 4, and 8 were used. As the TR was reduced with increasing MB factor, the flip angle was optimized to match the Ernst angle based on a grey matter (GM) T1 value of 1,000 ms at 3 T (Weiskopf et al., 2013). All multiband RF excitations were performed with MB RF Phase Scramble selected (Wong 2012) and the data were reconstructed using the MB LeakBlock Kernel option (Cauley et al., 2014), which has been shown to suppress residual aliasing of BOLD signal across slices in fMRI (Risk, Kociuba, & Rowe, 2018; Todd et al., 2016). The same echo‐time was chosen for all the protocols (TE = 30.2 ms). No in‐plane acceleration was used.

**Table 1:** Acquisition parameters

MB factor	1	2	4	8
CAIPI shift	X	FOV/2	FOV/3	FOV/3
Field of view [mm^2^]	192 × 192			
Image matrix	64 × 64			
Phase oversampling [%]	12			
Number of slices	40			
Slice orientation	Transverse (PE direction : AP)			
In‐plane voxel size [mm^2^]	3 × 3			
Slice thickness [mm]	2.5			
Slice gap [%]	20			
TE [ms]	30.2			
Flip angle [°]	87	76	60	45
TR [ms]	2,800	1,400	700	350
*N* (number of samples)	153	306	612	1,224

### 3.2 fMRI processing

Each time series was realigned to the first volume and co‐registered to a T1‐weighted image acquired in the same scanning session. The unified segmentation algorithm (Ashburner & Friston, 2005), as implemented in SPM12, was used to generate participant‐specific GM masks and to normalize all data to Montreal Neurological Institute (MNI) group space. Volumes were then smoothed with a 6 mm FWHM isotropic Gaussian kernel. The design matrices of all GLMs included regressors for motion, a high‐pass filter (cutoff period 128 s), and the stimulation blocks convolved by the canonical hemodynamic response function. A set of 14 physiological regressors, generated using an in‐house developed Matlab toolbox (Hutton et al., 2011), were based on cardiac and respiratory traces recorded on Spike2 (Cambridge Electronic Design Limited, Cambridge, UK) with a respiration belt and pulse oximeter. Twelve regressors, based on a set of sine and cosine Fourier series components extending to the third harmonic, were built to model the cardiac and respiratory phase (Glover, Li, & Ress, 2000; Josephs, Howseman, Friston, & Turner, 1997). Two additional regressors were included to model the variation in respiratory volume (based on Birn et al., 2006, 2008) and heart rate (based on Chang & Glover, 2009) .

A cohort‐wise GM mask was defined as those voxels that had a GM probability >0.6 in at least half of the cohort. This mask defined the voxels included in the estimation of the GLM parameters. Activation based on viewing scenes or objects was expected in primary visual cortex (V1) and so a further participant‐specific mask of V1 was defined as described in a previous study (Todd et al., 2017).

Temporal autocorrelations were modeled either with a mixture of an AR(1) model + white noise or with the FAST model (SPM12 revision 7203) with varying numbers of components, $p\in \left[1\;9\right].$ For reference, the data were also analyzed without prewhitening.

### 3.3 Evaluation of the “FAST” model

#### 3.3.1 Efficiency of prewhitening

A Ljung‐Box Q test (Box & Pierce, 1970) was used to test if any autocorrelations remained in the residuals of the first 100 data points after estimating the parameters of the GLM. Every lag up to 20 volumes was tested. The proportion of voxels rejecting the null hypothesis, of no correlation at any lag, was calculated, with significance defined as *p* < .05 after false discovery rate correction for multiple comparison. Several GLMs were tested by including or excluding the 14 physiological regressors in the design matrix but also by varying the size of the dictionary of covariance components, 3*p*, with *p* ranging from 1 to 9.

#### 3.3.2 The stability of the estimator

The standard precision (inverse of the standard error) of the model parameter estimates is expected to increase linearly with the square root of the number of samples. This was assessed by truncating each time series. To ensure sufficient data was available to estimate the model parameters, the minimum number of samples was set to 100. The number of samples was therefore varied from 100 to 153*MB factor in 5 equal intervals. Given that the variation in the number of samples was too small (from 100 to 153) for the longest TR, this dataset was not analyzed.

For each of the three TRs, the temporal correlations were modeled with the FAST model using either 18 (*p* = 6) or 27 (*p* = 9) components. In addition, the optimal model as determined by the Ljung‐Box Q test was also examined. This analysis was performed with physiological regressors included in the design matrix. The model that was deemed optimal was the one that used the fewest model components while still resulting in the minimum temporal correlations in the residuals. This was FAST with 9 components for TR = 1.4 s, FAST with 12 components for TR = 0.7 s, and FAST with 15 components for TR = 0.35 s.

#### 3.3.3 Bayesian model comparison

The hyperparameters of the GLM model are estimated via restricted maximum likelihood (ReML) as implemented in SPM. In this context, the objective function is the (log) marginal likelihood as approximated by variational free energy. This accounts for the accuracy but also the complexity of the model of temporal correlations (Friston et al., 2007). The free energy provides a lower bound on the model evidence enabling Bayesian model comparison. For a given covariance component dictionary size, the ReML algorithm returns the hyperparameter values (i.e., covariance parameters: $\lambda_{i}$) that maximize the free energy. In this study, the resulting free energy (i.e., log marginal likelihood or model evidence) was compared across 10 dictionaries: the FAST model with $p\in \left[1,9\right]$ and the AR(1) + white noise model. As implemented in SPM (spm_reml.m), the algorithm used for the AR(1) + white noise model or the FAST model was exactly the same, with the exception that the number of hyperparameters was increased for the latter. The algorithm performs a Fisher scoring ascent on variational free energy (i.e., a lower bound on Bayesian model evidence) to identify maximum a posteriori covariance component (hyper) parameter estimates, as described in (Friston et al., 2002; Penny et al., 2007; Starke & Ostwald, 2017). The same priors as for the conventional AR(1) + white noise model were used for each hyperparameter, $\lambda_{i}$. Technically, this inversion scheme is referred to as variational Laplace because it assumes a Gaussian posterior over covariance component (hyper) parameters, that are equipped with an uninformative (hyper) prior, with a variance of exp(8).

A detailed description of the strategy used to evaluate the FAST model is provided as Supporting Information.

### 3.4 Evaluation of functional sensitivity measures

#### 3.4.1 Simulation: *t*‐score testing for the mean versus weighted tSNR

A simple simulation was carried out to illustrate the potential theoretical benefit of using the *t*‐score of the mean rather than the more typical weighted tSNR. Numerical 2D time series $S_{i}$ of 100 voxels and 1,024 temporal samples, indexed by *i*, were simulated with a mean signal of 100 and a BOLD‐related signal with an effect size of 1%. The BOLD‐related signal was composed of 16 blocks of 32 s, each separated by an interval of 32 s, and convolved by the HRF. Temporal correlations were simulated with an autoregressive model of order 1 and parameter 0.4 with a standard deviation of 1 and added to the time‐series of each voxel. To simulate the case of having a fixed total scan time but variable sampling interval, six time series $S_{i}^{d},\;d\in [1;6]$ were derived from this original series by selecting one sample every *d* samples. For example, $S_{i}^{2}$ has a sampling interval twice that of $S_{i}^{1}$ but has only half the number of samples. The parameters of the GLM of each time series were estimated via SPM with an AR(1)+white noise model of temporal correlation. The average of the *t*‐score testing for the mean signal and the *t*‐score testing for the simulated task signal were computed and compared.

#### 3.4.2 In vivo: *t*‐score testing for the mean versus weighted tSNR

The estimated model parameters and hyperparameters were used to compute the tSNR weighted by the number of samples $\left(\frac{c_{0}^{T}\hat{\beta }}{\hat{\sigma }}\sqrt{N}\right)$, the *t*‐score testing for the mean signal $\left(\frac{c_{0}^{T}\hat{\beta }}{\hat{\sigma }\eta_{0}}\right)$ and the *t*‐score testing for the contrast scenes versus objects. The first two metrics, based on the mean signal over time, were averaged within V1 for each participant whereas for the task‐based contrast, the 10% highest *t*‐scores for each participant were averaged within V1. To summarize the results across the cohort, the median and interquartile range of each metric were subsequently calculated across participants. This analysis was carried out with the conventional AR(1) + white noise model, the FAST model with 18 components and the optimal model as selected by the Ljung‐Box Q test. The FAST model with 18 components has been specifically tested here because this is the model with the highest number of components showing the maximum free energy for at least one participant. A model with a greater number of components was never selected.

## 4 RESULTS

### 4.1 Evaluation of the FAST model

#### 4.1.1 Efficiency of prewhitening

**Figure 2:**
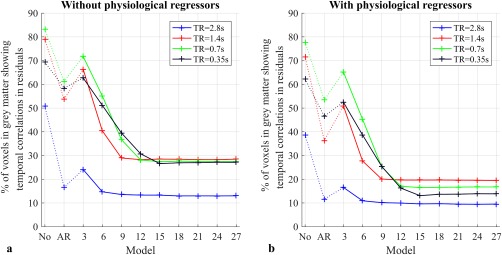
Proportion of voxels showing temporal correlations in the residuals of the GLM (without (a) and with (b) physiological regressors included in the design matrix). Each data point is the average across all the participants. The Ljung‐Box *Q* test is performed with 11 different models for temporal correlations including the conventional AR(1)+ white noise model (AR), no temporal correlations (No), and the FAST model with *p* varying from 1 to 9. The significance level is defined as *p* < .05 after false discovery rate correction [Color figure can be viewed at http://wileyonlinelibrary.com]

The efficiency of the prewhitening step and thereby the accuracy of the auto‐correlation matrix *V* was characterized via the results of the Ljung‐Box Q test. The null hypothesis is that there are no temporal correlations, up to 20 volumes of lag, in the first 100 data points of the residual time‐series. Figure 2 shows the proportion of voxels from within the gray matter mask rejecting the null hypothesis with a maximum false discovery rate of 0.05. The test is performed on the residuals of the GLM with a design matrix including (Figure 2a) or not (Figure 2b) the physiological regressors.

- With the longest TR of 2.8 s, the proportion of voxels for which the null hypothesis was rejected was 50.7%, when no physiological regressors or prewhitening step was included. This proportion reduced to 38.6% if the physiological regressors were included in the design matrix. Prewhitening the time‐series with an AR(1) + white noise model reduced this proportion to 16.5% and 11.5%, without and with physiological regressors, respectively. The FAST model with 3 components is not as efficient as the AR(1) + white noise model given that the proportion of voxels with remaining temporal correlations was 24.1% and 16.5% without and with physiological regressors, respectively. However, using the FAST model with at least 6 components reduced the level of residual correlations below that achieved with the AR(1) + white noise model, to <14.7% and <10.9% without and with physiological regressors, respectively.
- With a TR of 1.4 s, the proportion of voxels for which the null hypothesis was rejected, without physiological regressors or prewhitening, reached 78.9%. This proportion was reduced to 71.6% by including physiological regressors in the design matrix. Additionally, prewhitening with the AR(1) + white noise model further reduced the proportion of voxels rejecting the null hypothesis to 36.3%. Although, the FAST model with 3 components did not reduce the proportion of voxels to the level achieved with the AR(1) + white noise model, 6 components improved the prewhitening performance (40.4% and 27.7% without and with physiological regressors, respectively). However, a minimum of 9 components was necessary in both cases (i.e., with or without physiological regressors) to reach a plateau level of 28.9% and 20.0% of voxels with remaining temporal correlations, respectively.
- The proportion of voxels for the TR of 0.7 s was the largest when no pre‐whitening step was used (83.1% without, and 77.7% of voxels with physiological regressors). The AR(1) + white noise model only reduced this proportion of voxels to 61.1% and 53.3%, respectively, whereas a plateau of 17.0% (28.1% without physiological regressors) was reached when the FAST model was used with 12 or more components.
- With the shortest TR of 0.35 s, the proportion of voxels rejecting the null hypothesis was 69.3% without prewhitening and decreased to 62.1% with physiological regressors. The AR(1) + white noise model further reduced this proportion to 46.5%. However, the FAST model with a minimum of 15 components was necessary to reach a plateau level of 13.1% with physiological regressors (27.0% without).

In summary, as the TR decreased, a greater number of model components was required to accurately prewhiten the time series.

**Figure 3:**
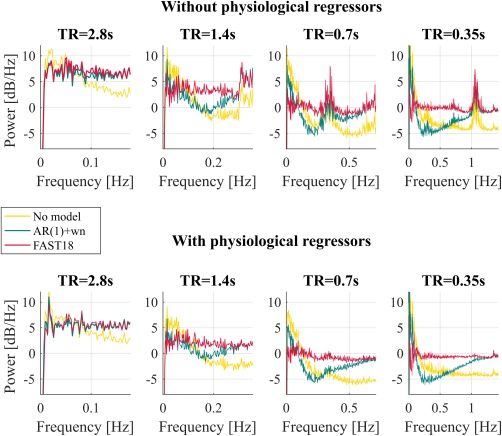
Power spectra of the residuals after fitting the GLM on the four time‐series (TR = 2.8, 1.4, 0.7, and 0.35 s) acquired on one exemplar participant. The design matrix did (bottom line) or did not (top line) include the physiological regressors. Two different models for temporal correlations were tested: AR(1) + white noise (blue curve) and FAST with 18 components (red curve) and compared to the residuals obtained without prewhitening (“No Model,” yellow curve) [Color figure can be viewed at http://wileyonlinelibrary.com]

The frequency content of the residuals was also calculated to further assess the relative performance of the AR(1) + white noise model and the FAST model with 18 components (Figure 3).

- The power spectra obtained without including physiological regressors in the design matrix showed frequency peaks consistent with physiological effects, regardless of which model for temporal correlations was used (Figure 3, upper row). These peaks were removed when physiological regressors were included in the design matrix.
- The power spectra showed low frequency contents at every TR when no prewhitening was applied. The residuals obtained with the AR(1) + white noise model with the longest TR (2.8 s) show a flat spectrum, whereas this is not the case for TR≤ 1.4 s.
- While the power spectra of the residuals obtained with the AR(1) + white noise model showed slow variation in power at low frequencies for TR ≤ 1.4 s, this was no longer present when using the FAST model.

#### 4.1.2 Stability of the model

- Figure 4 shows the relationship between the standard precision of the parameter estimate for the constant term of the GLM (i.e., the mean signal) and the square root of the number of samples calculated for time‐series acquired with TR of 1.4, 0.7, and 0.35 s with different dictionary sizes for the covariance components in the FAST model. As expected, a linear correlation (*R* ^2^ ≥ 0.96) was observed for the dictionary sizes deemed optimal by the Ljung‐Box Q test, that is, the minimum number of components that achieves minimum residual temporal correlation: 9 for TR = 1.4 s, 12 for TR = 0.7 s, and 15 for TR = 0.35 s.
  **Figure 4:**
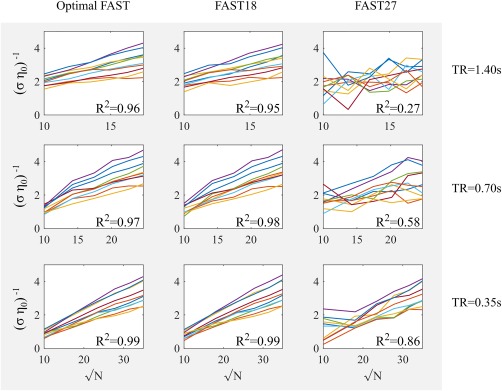
Relationship between the standard precision (inverse standard error) of the parameter of the constant term and the number of samples. Temporal correlations were modeled with 9, 18, and 27 components for TR = 1.4 s; with 12, 18, and 27 components for TR = 0.7 s; and with 15, 18, and 27 components for TR = 0.35 s. The coefficient of determination *R* ^2^ of the linear regression averaged across participants is indicated for each TR and model. Each color represents one participant [Color figure can be viewed at http://wileyonlinelibrary.com]
- The same relationship was observed using a FAST model with 18 components for every TR (*R* ^2^ ≥ 0.95).
- However, a large deviation from the linear correlation was observed for all TRs when the dictionary size was 27. The deviation was larger for longer TR (*R* ^2^ = 0.27, 0.58, and 0.86 for TR = 1.4, 0.7, and 0.35 s, respectively). This effect is due to poorly conditioned covariance components and subsequent numerical instabilities.

#### 4.1.3 Complexity and accuracy of the model

**Figure 5:**
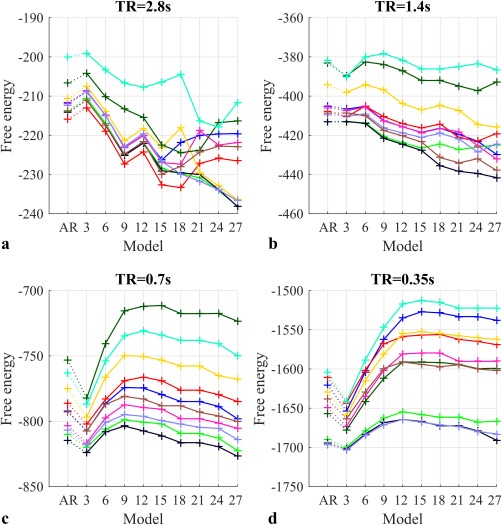
The resulting free energy for each model (abscissae), each participant (colors), and each TR: (a) 2.8 s; (b) 1.4 s; (c) 0.7 s; (d) 0.35s [Color figure can be viewed at http://wileyonlinelibrary.com]

The free energy was calculated for each participant and each prewhitening model of temporal correlations (Figure 5).

- With the longest TR of 2.8 s, the maximum free energy was obtained for the FAST model with 3 components for every participant. However, for every participant, the difference in free energy between the AR(1) + white noise model and the FAST model with 3 components did not exceed 3, a minimum threshold commonly used in Bayesian analyses to define a significant difference in evidence for one model over another (i.e., a log odds ratio of $e^{3}\approx 20:1$) (Jeffreys, 1961).
- For a TR of 1.4 s, the number of dictionary components that maximized the free energy varied across participants: free energy was maximized with AR(1) + white noise model for 2 participants, FAST with 3 components for 2 participants, FAST with 6 components for 5 participants, and FAST with 9 components for 1 participant. However, none of these models showed a difference in free energy relative to the AR(1) + white noise model that exceeded 3.
- The dictionary sizes that maximized the free energy increased as the TR was reduced to 0.7 s: FAST with 9 components for 7 participants, FAST with 12 components for 2 participants, and FAST with 15 components for 1 participant. In this case, all these FAST models showed a difference in free energy relative to the AR(1) + white noise model that was greater than 3, indicating a significant difference in model evidence in favor of the FAST model.
- With the shortest TR of 0.35 s, the maximum free energy was produced by the FAST model with 12 components for 4 participants, FAST with 15 components for 4 participants, and FAST with 18 components for 2 participants. Again, all these FAST models showed a difference in free energy, relative to the AR(1) + white noise model, greater than 3.

### 4.2 Evaluation of functional sensitivity measures

#### 4.2.1 Simulation: t‐score testing for the mean versus weighted tSNR

**Figure 6:**
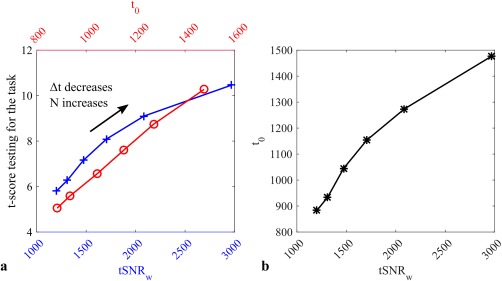
(a) Simulated relationship between the *t*‐score testing for the task and the weighted tSNR (tSNR_w,_ blue cross) or the *t*‐score testing for the mean signal (*t*_0_, red circle). By increasing the number of samples while decreasing the sampling interval, the weighted tSNR overestimates the increase in functional sensitivity, whereas the *t*‐score testing for the mean is directly proportional to the functional sensitivity. Indeed the *t*‐score testing for the mean signal and the weighted tSNR are not proportional (b), instead the *t*‐score testing for the mean tends to increase less rapidly than the weighted tSNR as the sampling interval decreases [Color figure can be viewed at http://wileyonlinelibrary.com]

Simulations show that when temporal correlations are accurately modeled, the *t*‐score testing for the task is directly proportional to the *t*‐score testing for the mean signal (Figure 6a, red) for all sampling intervals examined. Conversely, as the sampling interval, Δt, decreases and the total number of samples, *N*, concomitantly increases, the tSNR weighted by the square root of *N* increases more rapidly than the *t*‐score testing for the task (Figure 6a, blue). While the *t*‐score testing for the mean signal is proportional to the weighted tSNR measure for low *N* and longer Δt, it increases less rapidly than the tSNR weighted by the square root of the number of samples as Δt decreases and *N* concomitantly increases (Figure 6b).

#### 4.2.2 In vivo: *t*‐score testing for the mean versus weighted tSNR

**Figure 7:**
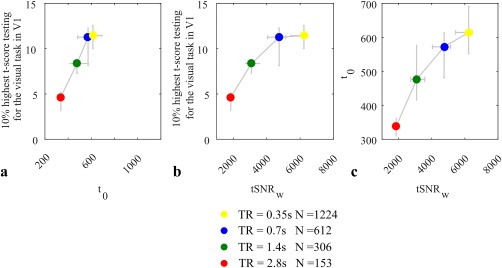
Relationship between the 10% highest *t*‐scores testing for the task (scenes vs object) in V1 and both the *t*‐score testing for the mean signal, $t_{0}$ (a) and the weighted tSNR, $tSNR_{w}$ (b) averaged across V1. Each data point is the median across participants. The vertical and horizontal bars illustrate the first and third interquartiles across participants. (c) The relationship between the *t*‐score testing for the mean signal and the weighted tSNR. The optimal model determined by the Ljung‐Box Q test is used for the prewhitening to remove temporal correlations in the time series [Color figure can be viewed at http://wileyonlinelibrary.com]

The *t*‐score testing for the visual task, the weighted tSNR, and the *t*‐score testing for the mean signal were computed across V1 using the optimal model as determined via the Ljung‐Box Q test: AR(1) + white noise model for the longest TR of 2.8 s, the FAST model with 9 components for TR = 1.4 s, the FAST model with 12 components for TR = 0.7 s, and the FAST model with 15 components for TR = 0.35 s. In agreement with the simulation results, a linear correlation between the average 10% highest *t*‐scores testing for the task and the average *t*‐score of the mean in V1 was observed, with a coefficient of determination of 0.99 (Figure 7a). This behavior was not observed for the weighted tSNR (Figure 7b), which instead tended to overestimate the benefit of increasing the number of samples by decreasing the volume acquisition time (TR). The relationship between the *t*‐score testing for the mean signal and the weighted tSNR observed in vivo (Figure 7c) was similar to that observed with the simulated data. The increase in weighted tSNR observed when the sampling interval was decreased from 2.8 to 0.35 s, thereby increasing the number of samples from 153 to 1,224 was 50% larger than the increase in the *t*‐score testing for the mean signal.

**Figure 8:**
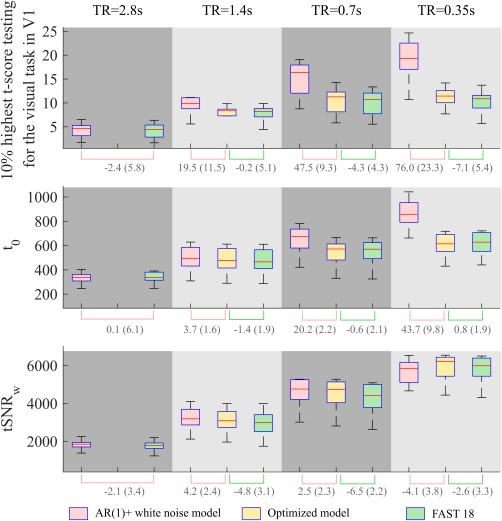
Variation of the 10% highest *t*‐scores testing for the task in V1, the averaged weighted tSNR ( $tSNR_{w})$ across V1, and the averaged *t*‐score testing for the mean signal ( $t_{0})$ across V1 with respect to the sampling interval (i.e., volume TR), the number of samples, and the model used to account for temporal correlations. Box plots represent the median and the interquartile range of the metrics across participants. Below the graphs, the median (interquartile range) of the percent difference between models are indicated: AR(1) + white noise with respect to the optimal model (pink line) and the FAST model with 18 components with respect to the optimal model (green line) [Color figure can be viewed at http://wileyonlinelibrary.com]

Modeling the temporal correlations, either with the AR(1) + white noise model or the FAST model with the appropriate number of components, made a great difference to the resulting *t*‐score (Figure 8) for the same statistical threshold.

- As shown in Figure 8, the *t*‐score testing for the task for TR = 1.4 s and *N* = 306 was 19.5% higher when calculated with the AR(1) + white noise model as opposed to the optimal FAST model. As the TR was reduced, thereby increasing the number of samples, this difference increased. At a TR of 0.35 s, producing 1,224 samples in the same scan duration, the *t*‐score was 76% higher when the AR(1) + white noise model was used as opposed to the more accurate FAST model.
- The weighted tSNR was also lower when using the optimal FAST model instead of the AR(1) + white noise model; however, this difference never exceeded 5% (relative to the optimal model).
- As opposed to the weighted tSNR, the *t*‐score testing for the mean was highly dependent on the model used to remove temporal correlations. Using the AR(1) + white noise model artificially increased the *t*‐score testing for the mean by as much as 43.7% relative to the optimal FAST model.

The results obtained with the optimal FAST model were also compared to those obtained with the FAST model with 18 components, which was the highest number of model components selected by Bayesian model comparison in the cases investigated in this study (selected for 2 participants when using a TR of 0.35 s). Percentage of difference was calculated with respect to the optimal model.

- The optimal FAST model and FAST with 18 components produced largely equivalent *t*‐scores when testing for the task. The maximum difference (7.1%) was observed for the shortest TR.
- The difference in weighted tSNR, between the analyses with the optimal FAST model and FAST with 18 components, was always less than 7%.
- The difference between the *t*‐score testing for the mean signal calculated with the FAST model using 18 components or the optimal FAST model was always below 2%.

## 5 DISCUSSION

Functional MRI analyses typically rely on a statistical test to infer BOLD‐related activation based on calculated *t* scores, using the Student *t* test. It can be seen from Equation 4 that the *t* score is the ratio of the GLM parameter estimates and their standard error. Therefore, in addition to the model estimates, the standard deviation of the time series and the correlation matrix of the observation error must also be calculated accurately. As such, the *t* score is dependent on several estimators, none of which should be neglected. The *t* score increases with the number of samples—via a decrease in the standard error of the parameter estimates, $\hat{\sigma }\eta$. However, increasing the number of samples without increasing the total acquisition time is only possible if the volume acquisition time (TR) is reduced. As illustrated by the Ljung‐Box Q test analyses (Figure 2a), temporal correlations in the error term occur more frequently when the TR is shorter (50% of voxels showed temporal correlations in the error term when TR = 2.8 s, whereas at least 69% of voxels showed this property when TR ≤ 1.4 s). This effect will clearly have an impact on the standard error of the parameter estimates and consequently on the *t*‐score testing for the contrast of interest. If the degree of temporal correlations is not correctly modeled, the standard error will be erroneous and preclude reliable inferences about any neuronal activation driving observed signal changes.

A simple way to remove long‐range temporal correlations is to apply a high‐pass filter to the data. All the results here were processed with a standard processing pipeline, including a high‐pass filter with a cutoff at 128 s, which has previously been shown to be an essential processing step (Worsley & Friston, 1995).

A second solution to account for temporal correlations is to regress out the physiological components of the signal that may drive serial correlations. Here, we have shown that this reduces the level of temporal correlations in the residuals regardless of the sampling interval (Figure 2b). The efficiency of the regression may depend highly on the way the regressors are generated, which in this study were derived from breathing and heart rate data recorded via respiratory belt and a pulse oximeter, respectively. The basis sine and cosine Fourier series modeling the fluctuations are sampled at the middle of the volume acquisition time, meaning that longer TRs will compromise their explanatory power, potentially reducing accuracy. However, the fact that frequency peaks consistent with physiological effects of cardiac pulsation and breathing were no longer visible in the spectra of the residuals (Figure 3) after including physiological regressors in the design matrix suggests that these effects have been modeled accurately. Nonetheless, the power spectra of the residuals continued to show structure at low frequencies that would be consistent with the hypothesis that unmodeled neural activity can also lead to serial correlations in the residuals (Bianciardi et al., 2009; Bollmann et al., 2018; Tong & Frederick, 2014).

As high‐pass filtering and the inclusion of physiological regressors do not fully remove temporal correlations, particularly at short TR (see Figure 2b, “No” condition and Figure 3, bottom line, “No model,” yellow curve), an additional—commonly used—step is to prewhiten the data before estimating the parameters of the GLM. This whitening procedure relies on having an appropriate model of the temporal correlations present in the data to accurately estimate the noise covariance matrix. The typical approach for fMRI studies is to use a mixture of white noise and a first‐order autoregressive process. By adding this third step, the proportion of voxels showing temporal correlations in the residuals further reduced to 11.5% with the longest TR. However, if the model fails to faithfully capture the temporal correlations, as appears to be the case here for TR ≤ 1.4 s (Figures 2 and 3), serial correlations will remain in the residuals and the *t* score used for inference will be overestimated, thereby increasing the false‐positive rate. The failure of the AR(1) + white noise model is also qualitatively observed in the power spectra of the residuals after prewhitening, which showed variation in power across frequencies for TR ≤ 1.4 s.

### 5.1 Investigation of the FAST model

The FAST model aims to more accurately model the temporal correlations in rapidly sampled data by incorporating a more complete model of temporal correlation with a larger number of components. This model offers the possibility of capturing temporal correlations at longer temporal lags. Combined with physiological regressors, this model reduced the proportion of voxels with temporal correlations in the residuals to below 20% regardless of the sampling interval (Figure 2), as long as the number of components was sufficiently high. The minimum number of components for a TR of 1.4 s was 9, whereas 12 components were required for TR of 0.7 s and 15 for TR of 0.35 s.

It must be noted that at short TR the proportion of voxels with remaining temporal correlations did not fall to the level achieved with a TR of 2.8 s, even when a large number of components was included in the FAST model, indicating that the shape of the covariance components within the dictionary may be insufficient to capture these remaining temporal correlations or that the use of a single global covariance matrix was not suitable for all voxels. For TR ≤ 1.4 s, the plateau‐level percentage of voxels in which temporal correlations remained decreased as the TR was shortened. This may reflect the fact that the SNR also fell such that thermal noise was increasingly prevalent.

The proportion of voxels with residual temporal correlations plateaued after a certain number of components were included in the model. The minimum number of components required to reach this plateau level increased as the TR decreased. As a Bayesian model reduction is already implemented in the ReML procedure (to remove unnecessary components), a naïve solution would be to use a very high number of components regardless of the sampling interval. However it appears that a very high number of components may not be suitable (Figure 4). A model including 27 components has been tested for TR ≤ 1.4 s. Although the remaining temporal correlation was very low according to the Ljung‐Box Q test results, the standard precision did not increase linearly with the square root of the number of samples when the time‐series was truncated. This behavior may reflect an overparameterization of the model resulting in an ill‐conditioned inversion problem; that is, severe conditional dependencies between the covariance component (hyper) parameters that render the posterior covariance rank‐deficient. The resulting overparameterization can be finessed by limiting the number of components used in the FAST model to ensure stable convergence. In principle, overparameterization can be compounded by inefficient sampling of serial correlations. This might occur, because SPM pools samples of serial correlations over voxels that survive an omnibus F‐test on task‐related regressors. This means inefficient task designs could lead indirectly to inefficient estimates of serial correlations—by limiting the number of voxels contributing to the estimators.

The free energy approximation to log model evidence (provided by the ReML algorithm) penalizes the complexity of the model. The optimal model would therefore provide the maximum free energy. This maximum was reached for different models depending on the TR. Based on this free energy model comparison, the optimal model for long TR (2.8 s) was the FAST model with 3 components. However, given that the difference in free energy was less than 3, we can conclude that there is no strong evidence for the use of FAST with 3 components over the AR(1) + white noise model in this case. However, for shorter TR, the minimum number of model components providing the maximum free energy increased with decreasing TR, consistent with the need to have more complex models of serial correlations in more rapid imaging scenarios. When using FAST the optimal number of terms was participant‐specific ranging from 3 to 9 for TR = 1.4 s, from 9 to 15 for TR = 0.7 s, and from 12 to 18 for TR = 0.35 s. These results are consistent with those of the Ljung‐Box Q Test.

Although computationally expensive, this framework (including the Ljung‐Box Q‐test, the analysis of the behavior of the standard precision as the number of samples is varied, and the free energy model comparison) would ideally be applied to every pilot study to choose the optimal model for removing temporal correlations with a given imaging setup (e.g., acquisition scheme and volume TR). Temporal correlations may vary depending on the pulse sequence used or the task involved. However, our analyses have additionally shown that the FAST model with 18 components (as implemented by default in SPM12, R7203) can be reliably used, at least for the conditions tested (i.e., TR ≥ 0.35s, block design). Indeed, based on the Ljung‐Box Q test, this dictionary size provides the minimum remaining temporal correlations regardless of the sampling interval while guaranteeing a linear correlation between the standard precision and the square root of the number of samples. Qualitatively, the residuals obtained using FAST with 18 components did not show any structure in the frequency spectra regardless of the TR considered. Furthermore, the difference in the *t*‐score testing for the visual task between the optimal FAST model and FAST with 18 components (Figure 8) never exceeded 7.1%.

As highlighted by previous studies (Bollmann et al., 2018; Eklund et al., 2012; Olszowy et al., 2017), the traditional AR(1) + white noise model may fail to prewhiten the data for short TR. Here, we have shown that a more comprehensive model can improve the efficiency of prewhitening for TR ≤ 1.4s. However, like the AR(1) + white noise model, the FAST approach still uses a global correlation matrix V. There may be additional benefit to be gained from using spatially varying model coefficients (Eklund et al., 2012; Penny, Kiebel, & Friston, 2003; Sahib et al., 2016). However, regionally specific estimates of serial correlations are necessarily less efficient and might introduce unwanted variability in the estimates of nonsphericity.

### 5.2 *t*‐score testing for the task, the mean signal, and the weighted tSNR

Note that the impact of temporal correlation modeling has only been investigated here for single‐subject analyses. When making inferences at the group level (using the standard summary statistic approach to random effects analysis), only (contrasts of) parameter estimates are taken to the second level, and not the standard error. As the model parameters are unbiased maximum likelihood estimators (and only the estimate of the standard error depends on serial correlations), serial correlations cannot bias inference at the group level when used in this context. The impact of serial correlations modeling for alternative analysis approaches that do propagate error estimates to the second‐level would require further investigation.

In first‐level analyses, the *t*‐score testing for the task is highly sensitive to the approach used to model temporal correlations. In this particular study, at a TR of 0.35 s, the *t*‐score testing for the visual task was increased by 75% compared to the optimal FAST model, reducing the proportion of voxels with residual temporal correlations to below 14%.

Our numerical simulations illustrate that, when temporal correlations are accurately modeled, the *t*‐score testing for the mean signal will provide a more accurate predictor of task‐related functional sensitivity than the tSNR, even when the latter is adjusted to account for the number of acquired samples (Figure 6). This was also confirmed by the *in vivo* analyses. Once the temporal correlations were properly modeled and taken into account, the *t*‐score of the mean was a better predictor of the functional sensitivity. The weighted tSNR does not account for temporal correlations and therefore tends to overestimate the functional sensitivity when the number of samples is increased (Figure 7). For example, the benefit of a high multiband factor of 8 over a lower factor of 4 was overestimated by the weighted tSNR. The *t*‐score testing for the mean signal indicates that the concomitant effects of increasing the g‐factor, reducing the flip angle and reducing the TR, lead to a tSNR decrease and more temporal correlations in the data, which counterbalanced the benefit of higher statistical power afforded by having more samples in the MB factor 8 case.

Although the weighted tSNR does not account for temporal correlations, Figure 8 shows a small variation in weighted tSNR across the different models of serial correlations. This is because it is estimated within SPM's GLM framework meaning that the estimate of the variance in the time series will in part depend on the model of temporal correlations. An unbiased estimate of the standard deviation requires accurate estimation of the correlation matrix *V* (Equation 5). If this is incorrectly taken to be the identity matrix, the estimator of the standard deviation is biased and underestimated (Zieba, 2010), the higher the degree of temporal correlation, the greater the underestimation. This also demonstrates that the chosen model affects not only the η parameter but also the standard deviation σ estimation, two key parameters of the statistical tests used in fMRI.

The computation of the *t*‐score testing for the mean signal is recommended as an alternative measure to be used when selecting a protocol. The input data may come from the pilot of the study or a simple task‐free acquisition. In the latter case, all voxels within the specified mask are used for estimating the covariance matrix, whereas only voxels correlating with the experimental conditions are used if such conditions are specified in the design matrix. Given that this measure includes the estimates of both σ and η, it should be more representative of the true functional sensitivity, as evidenced by the results presented here.

A key insight from this analysis is that improving the sensitivity of fMRI is not simply a matter of reducing the amplitude of random fluctuations, which in any event may be irreducible if they are physiologically mediated. Rather it is important to characterize how efficiently both the amplitude of the signal and any random fluctuations can be estimated. In other words, one has to accurately quantify the uncertainty about the parameter estimates. Given that the functional sensitivity is also dependent on the covariance of the design matrix, which will further increase η, that is, increase the standard error of the parameter estimate, the *t*‐score testing for the mean may concurrently help in selecting both the optimal sequence and the optimal task design.

## 6 CONCLUSION

Temporal correlations are a crucial feature of the time‐series acquired in fMRI experiments and must be accurately modeled to avoid overestimation of the *t*‐score and unreliable statistical inferences leading to increased false‐positive rates. The traditional AR(1) + white noise model for temporal correlations is susceptible to failure at short TR. A more complete model, implemented as the “FAST” option in SPM, has been designed to capture temporal correlations with longer temporal lag and has proven to be more powerful for TR ≤ 1.4 s. Comparing the functional sensitivity of sequences with different numbers of samples, sampling intervals, and SNR is not trivial, especially because currently used metrics do not account for temporal correlations. To address this issue, the *t*‐score testing for the mean is proposed as an alternative to the weighted temporal SNR. This metric shows higher correlation with functional sensitivity as long as an accurate model for temporal correlations is used.

## Supporting information

Additional Supporting Information may be found online in the supporting information tab for this article.

Supporting Information
