# Supplementary material for: Accurate modeling of temporal correlations in rapidly sampled fMRI time series
Source: Hum Brain Mapp. 2018 Jun 8;39(10):3884–97. doi: 10.1002/hbm.24218 (PMC6175228; doi:10.1002/hbm.24218)
Supplement: Supplementary file 1 — Supporting Information [file HBM-39-3884-s001.docx]

Supplementary Material

This appendix provides a step-by-step guide to implementing the strategy that has been used to evaluate the performance of a model of temporal correlations. The presented framework is flexible and could therefore be used to test any aspect of the model, e.g. the form of the covariance components. In the present study, an AR(1) + white noise model was compared with the FAST model, which in turn was implemented with a variable numbers of components. The best model among those examined can be expected to show:

- The minimum number of voxels with temporal correlations remaining in the residual time-series.
- A linear correlation between the standard precision and the square root of the length of the time-series.
- The maximum free energy.
- ***The Ljung-Box Q test***

This tests the null hypothesis that no temporal correlations are present in the data. The best model is that which rejects the null hypothesis in the fewest voxels.

**Protocol:**

1. Write the residuals when estimating the GLM with a given model (an SPM option)
2. Apply the Matlab function lbqtest.m to the residuals of the time-series of every voxel of interest, testing for lags up to 20 volumes by default.

| **Code 1:** Ljung-Box Q test |
| --- |
| **Input:** voxel-specific residual time-series, y(v,t)  **Output:** voxel-specific p-values, p(v), for the hypothesis test    **for** all voxels indexed by v  [~,p(v)]=lbqtest(y(v,:))  **End** |

1. Having applied an adequate correction for multiple hypothesis testing, calculate the percentage of voxels that have a p-value below the threshold used to denote statistical significance.
2. The optimal model has the lowest percentage.

- ***The standard precision of the mean***

A model that does not show a linear correlation between the standard precision and the square root of the number of samples in the time-series, for a constant temporal resolution, should be rejected.

**Protocol:**

1. Repeatedly estimate the GLM parameters while increasing the length of the time-series, but keeping the temporal resolution constant.
2. Compute the standard precision of the mean for each time series.

| **Code 2:** Estimating the standard precision of the mean |
| --- |
| **Input:** SPM structure from the SPM.mat file  *VResMS* Matrix containing the residual variance (obtained from the nifti file ResMS.nii)  **Output:** Standard precision *Sp* of the mean   1. Create the contrast vector for the mean (contained in the final column of the design matrix)   c=zeros(size(SPM.xX.X,2),1);  c(end)=1;   1. Estimate the $\eta$(eta) parameter   eta=sqrt(1./(c'*SPM.xX.Bcov*c));   1. Calculate the standard precision of the mean Sp   Sp=1/(VResMS*eta) |

1. Calculate the correlation between the standard precision and the square root of the length of the time-series.

- ***The free energy of a given model***

The free energy metric is used within SPM to estimate the hyperparameters. The hyperparameters providing the highest free energy are selected. A slight modification of the SPM code allows one to retrieve the final free energy of a given model and compare it to another model.

**Protocol:**

1. Return the free energy

| **Code 3:** Return the free energy |
| --- |
| **Input:** SPM structure from the SPM.mat file  *VResMS* Matrix containing the residual variance (obtained from the nifti file ResMS.nii)  **Output:** Free energy, F  Accuracy component of free energy, Fa (optional)  Complexity component of free energy, Fc (optional)  **In the file:** spm_est_non_sphericity.m  **Replace**  [Vp,hp] = spm_reml(Cy(q,q),Xp,Qp);  **By**  [Vp,hp,Phres,F,Fa,Fc] = spm_reml(Cy(q,q),Xp,Qp); |

1. Compare the free energy values obtained for each model. The optimal model maximises the free energy.

- ***The number of components of the FAST model***

The parameters of the FAST model can be easily changed. While any aspect of the model can be investigated, in this study only the parameter *p* (c.f. Eq. 6) dictating the number of components included in the model has been changed. The procedure for investigating the impact of this parameter is detailed below.

| **Code 4:** Change the parameter p of the FAST model (by default p = 6) |
| --- |
| **In the file:** spm_Ce.m (R7203)  **Replace :**                  T     = (0:(v(m) - 1))*dt;                  d     = 2.^(floor(log2(dt/4)):log2(64));                  for i = 1:***min(6,length(d))***                      for j = 0:2                          QQ = toeplitz((T.^j).*exp(-T/d(i)));                          [x,y,q] = find(QQ);  …  end  end  **By**                  T     = (0:(v(m) - 1))*dt;                  d     = 2.^(floor(log2(dt/4)):log2(64));                  for i = 1: ***p***                      for j = 0:2                          QQ = toeplitz((T.^j).*exp(-T/d(i)));                          [x,y,q] = find(QQ);  …  end  end |

Note: for SPM versions prior to R7018, this code was located in the function spm_fmri_spm_ui.m and the default number of components was not capped at 6 but rather was fully dictated by the specified TR of the data (parameter “dt” in the above code snippet).
